# Comment on Fauconnier, A. Phage Therapy Regulation: From Night to Dawn

**DOI:** 10.3390/v11090771

**Published:** 2019-08-22

**Authors:** Eric Pelfrene, Zigmars Sebris, Marco Cavaleri

**Affiliations:** 1 Office of Anti-Infectives and Vaccines, Human Medicines Evaluation Division, European Medicines Agency, 1083 HS Amsterdam, The Netherlands; 2 Regulatory Affairs Office, Human Medicines Evaluation Division, European Medicines Agency, 1083 HS Amsterdam, The Netherlands

**Keywords:** antimicrobial resistance, bacteriophage, personalised medicines, phage therapy, pharmaceutical legislation, regulatory framework

With most interest we read the contribution made by Fauconnier on phage therapy regulation [1] and wish to complement it by expressing our personal view on the topic. Indeed, with the threat of antimicrobial resistance, bacteriophage therapy has received renewed attention in the Western world, as a potential viable alternative treatment option [2]. Nevertheless, as asserted by the author, the Europe-wide regulatory approval of such biological product is less than straightforward and might prove even more problematic for the cited tailor-made concoctions. We concur with the view that the current EU pharmaceutical legislation is not optimised to allow certain individualised medicines models, such as selecting active substances individually for each patient from a “library” or cultivating individualised bacteriophages in vitro in presence of pathogens obtained from the patient. Nor seems frequent and prompt updating of the active substance (i.e., changes of active substance of a product in response to emerging resistance) easy to accommodate. As such, we empathise with the author’s “call-to-arms” for a paradigm shift in the regulatory framework for bacteriophages. Nevertheless, it is stressed that cardinal scientific uncertainties in relation to bacteriophage therapy remain to be addressed. So far, apart from sparse available comparative data [3], contemporary evidence on successful bacteriophage deployment has mainly been obtained from a few reports, albeit definitely involving difficult-to-treat chronic infected and/or severely ill subjects [4,5], and with reported use in a singular instance of a genetically modified product as to enhance virulence [6]. However, lack of robust evidence from randomised clinical trials precludes definition of the therapeutic impact. Moreover, aspects such as site of infection and route of bacteriophage administration could prove to be important determinants for efficacy, immune responses and the potential for adverse events. Thus, to date, no conclusive evidence is available on whether the proposed novel approaches will ultimately prove to be safe and effective for general implementation. These considerations seem to have been largely ignored in the Opinion paper. It remains to be further elucidated which additional data will be required to demonstrate the quality, efficacy and safety of each bacteriophage variant (e.g., each entry in the “library” or modified bacteriophage in response to resistance). Identification of a suitable regulatory framework is impacted by the uncertainties in the evidence and in the actual development plans that could be put in place.

The European Medicines Agency (EMA) engages in constructive dialogue with the phage scientific community through workshops, symposia and via contributions to the scientific literature, as to determine such an appropriate EU-wide framework, allowing licensing and timely use of bacteriophages in specified conditions [7,8]. In the meantime, however, the Agency calls on the developers of bacteriophage therapeutics to expedite the availability of comparative data, addressing aspects of safety, efficacy and phage resistance potential in pursued indications. Such action will definitely foster further confidence in such therapeutics and largely facilitate any future adjustment to the EU regulatory environment in support of bacteriophages’ therapeutic use. The Agency also encourages discussion on any individual development plan as to explore options for a way forward, and to this effect, recently expanded the scope of the EMA’s Innovation Task Force framework specifically for developers who work on therapeutic approaches for the treatment or prevention of bacterial or fungal infections [9]. We acknowledge that new approaches for the authorisation of bacteriophages may result also from any future initiative regarding the EU-wide regulatory framework for personalised medicinal treatment. Ultimately though, any emerging framework will need to strike a right balance, meeting patients’ needs for customised personalised medicines, whilst also respecting societal expectations for proven quality, safety and efficacy of the products.
